# The Effects of Dietary Supplementation of *Saccharomyces cerevisiae* Fermentation Product During Late Pregnancy and Lactation on Sow Productivity, Colostrum and Milk Composition, and Antioxidant Status of Sows in a Subtropical Climate

**DOI:** 10.3389/fvets.2020.00071

**Published:** 2020-02-18

**Authors:** Jun Chen, Yufeng Zhang, Jinming You, Hanqing Song, Yinzhi Zhang, Yantao Lv, Hanzhen Qiao, Min Tian, Fang Chen, Shihai Zhang, Wutai Guan

**Affiliations:** ^1^Guangdong Provincial Key Laboratory of Animal Nutrition Control, College of Animal Science, South China Agricultural University, Guangzhou, China; ^2^Jiangxi Province Key Laboratory of Animal Nutrition, Engineering Research Center of Feed Development, Jiangxi Agricultural University, Nanchang, China; ^3^College of Animal Science and National Engineering Research Center for Breeding Swine Industry, South China Agricultural University, Guangzhou, China

**Keywords:** antioxidant status, colostrum and milk composition, *Saccharomyces cerevisiae* fermentation product, sow, subtropical climate

## Abstract

This study aimed to evaluate the effects of dietary supplementation of *Saccharomyces cerevisiae* fermentation product (SCFP) during late pregnancy and lactation on sow productivity, colostrum and milk composition, and antioxidant status of sows in a subtropical climate. The study was a 2 × 2 factorial treatment design where the first factor was environmental THI level [Low THI (08:00–19:00: 70.76 ± 0.45, 19:00–08:00: 67.91 ± 0.18, L-THI) or High THI (08:00–19:00: 75.14 ± 0.98, 19:00–08:00: 68.35 ± 0.18, H-THI], and the second factor was dietary treatment (supplemented with or without 3 kg/t SCFP). A total of 120 sows were randomly allotted to the four treatments (*n* = 30). The feeding trial was conducted from 85-days post-breeding until 21-days post-partum. Compared with L-THI group, sows from H-THI group had lesser individual piglet birth weight, individual piglet weight at weaning, preweaning average daily gain of piglets, average daily feed intake of sows during lactation, and protein percentage in 14-days milk. Additionally, sows from H-THI group had lesser antioxidant status, indicated by lesser serum total antioxidant capacity (T-AOC), and superoxide dismutase (SOD) activity at parturition; lesser serum T-AOC and glutathione peroxidase (GSH-Px) activity at 14-days post-partum, as well as lesser SOD activity in colostrum. Compared with sows fed the control diet, sows fed the SCFP diet had greater number of piglets weaned, litter weight at weaning, and preweaning average daily gain of piglets. Moreover, sows fed the SCFP diet had improved antioxidant status as indicated by higher serum T-AOC at parturition, and lesser malondialdehyde (MDA) content in colostrum and 21-days milk. In conclusion, H-THI negatively affected the productivity, milk composition, antioxidant status, and lactation feed intake of sows. Dietary supplementation of SCFP partially alleviated the adverse effects of H-THI, by improving lactation performance and antioxidant status of sows without influencing reproductive performance and colostrum and milk composition in a subtropical climate.

## Introduction

Over fifty percent of total world meat and 60% of total world milk production are produced in tropical and subtropical areas, and livestock production in these regions will continue to provide significant meat production in the future (1). Heat stress is a primary factor adversely influencing welfare and production efficiency of sows in hot weather (2–4), which results in substantial economic losses for the swine industry (5). THI (temperature-humidity index) is commonly used as an indicator of the degree of heat stress (2, 6, 7). Under heat stress conditions, sows usually exhibit decreased feed intake, reduced colostrum and milk yield, and reduced milk quality (8). It has been reported that sows under heat stress suffer from oxidative stress, especially perinatal sows, related to high metabolism required for the rapid growth and development of the fetus and mammary gland, and for colostrum and milk production (9–11).

*Saccharomyces cerevisiae* fermentation product (SCFP) is a fermentation product using an unmodified strain of *Saccharomyces cerevisiae*, which includes fermentation products, residual yeast cells, fermentation media, and yeast cell wall components (12). It's demonstrated that antioxidant additives are beneficial for livestock under stressful conditions (13–15). SCFP is a widely-used feed addictive characterized as having antioxidant function (16, 17), and anti-heat stress function (16, 18–21), and was reported to improve lactation performance and health status of dairy cows under heat stress conditions (16, 18–20). SCFP was also reported to improve lactation performance of sows (12, 22, 23). Kim et al. reported feeding SCFP (12 and 15 g/d during gestation and lactation, respectively) to sows during middle and late pregnancy and lactation increased litter weight gain by 6.9% (*P* < 0.01) (23). Kim et al. conducted another study involving 491 mixed-parity sows to explore the effects of feeding SCFP to sows during middle, late gestation and lactation on sow productivity, and they reported that SCFP supplementation increased litter weight gain for the multiparous sow (22). Similarly, Shen et al. reported the effects of supplementation of SCPF during whole gestation and lactation on sow and litter performance, and found that feeding 12 (gestation) and 15 g/d SCFP (lactation) to sows had no effect on reproductive performance of sows, but improved litter weight at weaning (*P* = 0.068) and litter weight gain (*P* = 0.084) (12). However, less is known about the effects of feeding SCFP to sows during late gestation and lactation on sow productivity, colostrum and milk composition, and antioxidant status under heat stress conditions.

Therefore, it is hypothesized that feeding SCFP to sows during the perinatal period can alleviate the negative impact of high THI as an indicator of heat stress, including poor lactation performance, colostrum and milk composition, and antioxidant status of sows in a subtropical climate.

## Materials and Methods

### Experimental Design

This study was carried out as a 2 × 2 factorial treatment design, in which the first factor was environmental THI level [Low THI (08:00–19:00: 70.76 ± 0.45, 20:00–09:00: 67.91 ± 0.18, L-THI) or High THI (08:00–19:00: 75.14 ± 0.98, 20:00–09:00: 68.35 ± 0.18, H-THI)], and the second factor was dietary treatment (supplemented with or without 3 kg/t SCFP, Diamond V Original XPC, Diamond V, Cedar Rapids, IA). A total of 120 sows (Land-race × Yorkshire, parity 3–8) were randomly allotted to four treatments according to historical reproductive performance, body condition and parity (*n* = 30). The feeding trial was conducted from 85-days post-breeding until 21-days post-partum, and then sows were transferred to a mating house, and the estrus rate to 7 days post-weaning was recorded. The feeding trial was carried out in a commercial pig farm in a subtropical climate region, Jiangmen City, Guangdong province in China, in summer, 2015. Sows in the L-THI group were reared in pad-fan cooling house both in late gestation and lactation, while sows in H-THI group were reared in a traditional house with natural ventilation. The temperature and relative humidity were recorded using an automated thermo-hygrometer (W-series, Wangyunshan, Fujian, China). The THI was calculated using temperature and relative humidity as parameters according to the method of Wegner et al. (3): THI = [(1.8T) + 32—[0.55(RH/100)] × [((1.8T) + 32)−58], in which T is temperature in °C and RH is relative humidity in %. The environmental parameters are shown in Table 1.

**Table 1:** Environmental parameters (Mean ± SE).

**Item**	**L-THI group**	**H-THI group**	***P*-value**
**08:00–19:00**			
THI	70.8 ± 0.451	75.1 ± 0.980	<0.001
Temperature (°C)	28.6 ± 0.313	31.0 ± 0.554	<0.001
Relative humidity (%)	91.8 ± 1.23	79.8 ± 2.41	<0.001
**20:00–09:00**			
THI	67.9 ± 0.182	68.4 ± 0.183	0.573
Temperature (°C)	26.3 ± 0.121	26.7 ± 0.140	0.468
Relative humidity (%)	98.6 ± 0.283	96.5 ± 0.511	0.296

*The data were analyzed using T-test of SPSS 22.0 software (SPSS, INC., Chicago, IL, USA)*.

*H-THI, high temperature-humidity index; L-THI, low temperature-humidity index*.

### Diets and Management

The experimental diets were corn and soybean-based diets with ingredient composition and nutritional levels listed in Table 2. The nutritional levels met or surpassed the nutritional requirements of sows during late pregnancy and lactation (24). The feeding trial was conducted from 85-days post-breeding until 21-days post-partum. During late pregnancy, all sows were fed 2.5~3.5 kg/d experimental diet according to their body condition. At 111-days post-breeding, sows were transferred to a farrowing house, and had *ad libitum* access to the experimental diets and water until 21-days post-partum (at weaning). Piglets were cross-fostered within treatments by 48 h post-partum, and litter sizes were adjusted to 10 ± 1 piglets. Piglets were provided creep feed at 7-days of age. After weaning, sows were transferred to a breeding house, and the estrus rate to 7 days post-weaning was recorded.

**Table 2:** Composition and nutrient content of basal diets (as-fed basis).

**Item**	**Composition**	**Item**	**Nutrient content**
Ingredient, g/kg		Calculated composition, unit	
Corn	584.8	DE, MJ/kg	14.31
Wheat bran, 15.7% CP	80.0	CP, g/kg	179.9
Soybean meal, 42.0% CP	240.0	CF, g/kg	31.5
Fish meal, 64% CP	20.0	Ash, g/kg	59.2
Palm oil	40.0	Fat, g/kg	68.2
Dicalcium phosphate	3.0	Ca, g/kg	10.2
Limestone	16.0	Total P, g/kg	7.9
Salt	3.0	Available P, g/kg	5.2
Sodium bicarbonate	2.0	Digestible Lys, g/kg	8.4
Sodium Sulfate	4.0	Digestible Met+Cys, g/kg	4.9
Vitamin and mineral premix^a^	3.0	Digestible Thr, g/kg	6.8
Choline chloride (50%)	2.0	Digestible Trp, g/kg	1.8
Vitamin C (95%)	0.2		
L-Thr	1.0		
Total	1000.0		

a *Vitamin and mineral premix supplied per kilogram of complete diet: 100 mg Zn (ZnSO_4_·H_2_O), 80 mg Fe (FeSO_4_·H_2_O), 25 mg Mn (MnSO_4_·H_2_O), 20 mg Cu (CuSO_4_·5H_2_O), 0.14 mg I (CaI_2_O_6_), 0.3 mg Se (Na_2_SeO_3_), 13,000 IU vitamin A, 4,000 IU vitamin D_3_, 30 IU vitamin E, 4 mg vitamin K_3_, 4 mg vitamin B_1_, 10 mg vitamin B_2_, 4.8 mg vitamin B_6_, 0.034 mg vitamin B_12_, 40 mg niacin, 20 mg D-pantothenate, 2 mg folic acid, 0.16 mg D-biotin*.

### Data and Sample Collection

#### Sow Productivity

At parturition, the reproductive performance data were recorded, including the number of total born, born alive, weak (birth weight >0.8 kg), healthy piglets, litter birth weight, and individual birth weight. With-in 48 h post-parturition, piglets per litter were adjusted within treatment group, and the litter size, litter weight, and individual weight were recorded after being cross-fostered. Average daily gain of piglets, survival rate of piglets, lactation average daily gain of sows, and the estrus rate to 7 days post-weaning were recorded.

#### Serum Sample

A subset of 6 sows was randomly selected and sampled for blood using ear venipuncture method at day 85 of pregnancy, and day 0 and 14 of lactation. After sampling, blood was kept at room temperature for 1 h, and then centrifuged at 3,500 rpm for 10 min. The serum was separated, transferred into micro-tubes, and stored at −80°C until analysis.

#### Colostrum and Milk Sample

Colostrum, 14-days milk and 21-days milk were sampled within 24 h post-parturition, and day 14 and 21 of lactation, respectively. Milk samples were collected after intramuscular injection of 20 IU oxytocin and stored at −80°C until analysis.

### Chemical Analysis

#### Colostrum and Milk Composition

The colostrum and milk composition including solids-not-fat, protein, lactose, and fat were analyzed via an automated milk analyzer (Milk-Yway-CP2, Beijing, China).

#### Antioxidant Status

Antioxidant status of serum, colostrum and milk were analyzed as described in our previous study (25–27) using commercially available kits (Nanjing Jiancheng Bioengineering Institute, Nanjing, China). The antioxidant status estimates included T-AOC, GSH-Px activity, SOD activity, GSH content, and MDA content.

### Statistical Analysis

Statistical analysis was conducted using General Linear Model procedure of SPSS 22.0 software (SPSS, INC., Chicago, IL, USA), arranged as a 2 × 2 factorial design with the THI level and dietary treatment being the main factors. The following model was used: *Y*_*ijk*_ = μ + *A*_*i*_ + *B*_*j*_ + *(AB)*_*ij*_ + *e*_*ijk*_, in which *Y*_*ijk*_ = dependent variable, μ = mean, *A*_*i*_ = THI level (*i* = L-THI or H-THI), *B*_*j*_ = dietary SCFP supplementation (*j* = yes or no), *AB*_*ij*_ = interaction effect between THI level and dietary treatment, *e*_*ijk*_ = random error. In case of a significant interaction, the significance of differences among treatments was detected using the Student Newman-Keuls Test. The estrus rate of sows during 7 days post-weaning and survival rate of piglets were analyzed as binomial traits (e.g., returned to estrus or not, survived or not) using chi-square test. Results were expressed as mean and SE except for the estrus rate of sows during 7 days post-weaning and survival rate of piglets as percentage. Probabilities <0.05 were regarded as significant, and probabilities >0.05 and <0.10 were regarded as tendencies among treatments.

## Results

### Reproductive Performance

The effects of dietary supplementation of *Saccharomyces cerevisiae* fermentation product during late pregnancy and lactation on reproductive performance of multiparous sows in a subtropical climate are shown in Table 3. Compared with L-THI, sows from H-THI had lower individual piglet birth weight (*P* < 0.10). Dietary supplementation of SCFP did not affect the reproductive performance of sows (*P* > 0.10), averaged over temperature-humidity index treatment. The THI × diet interaction influenced the number of piglets born, live piglets born, healthy piglets and individual piglet birth weight (*P* < 0.05). The estrus rate of sows during 7 days post-weaning was unaffected by experimental treatments (*P* > 0.10).

**Table 3:** Effects of dietary supplementation of *Saccharomyces cerevisiae* fermentation product during late pregnancy and lactation on reproductive performance of sows in a subtropical climate (Mean ± SE)^*^.

**Item**	**L-THI**		**H-THI**		***P*****-value**		
	**Control diet**	**SCFP diet**	**Control diet**	**SCFP diet**	**THI**	**Diet**	**THI × Diet**
*N*	30	30	30	30			
Total no. of pigs born/litter	12.2 ± 0.501^a^	11.1 ± 0.463^ab^	10.7 ± 0.471^b^	12.8 ± 0.424^a^	0.824	0.234	0.001
No. of live pigs born/litter	10.6 ± 0.362^ab^	10.5 ± 0.221^ab^	10.0 ± 0.494^b^	11.8 ± 0.405^a^	0.209	0.183	0.003
No. of healthy pigs	10.3 ± 0.333^ab^	10.1 ± 0.423^ab^	9.6 ± 0.462^b^	11.3 ± 0.383^a^	0.251	0.164	0.005
No. of weak pigs/litter	0.321 ± 0.121	0.396 ± 0.101	0.410 ± 0.160	0.561 ± 0.182	0.115	0.917	0.321
Litter birth weight (kg)	15.5 ± 0.550	15.5 ± 0.741	15.0 ± 0.681	16.6 ± 0.583	0.644	0.215	0.215
Individual piglet birth weight (kg)	1.48 ± 0.041^ab^	1.58 ± 0.042^a^	1.52 ± 0.041^a^	1.41 ± 0.031^b^	0.095	0.894	0.007
Estrus rate of sows during 7 days post-weaning (%)	90.0	96.7	86.7	86.7	0.224	0.543	NA

*H-THI, high temperature-humidity index; L-THI, low temperature-humidity index, NA, not available*.

* *Results were expressed as mean and SE except for the estrus rate of sows during 7 days post-weaning as percentage. Values within a row with different superscripts differ significantly at P < 0.05*.

### Lactation Performance

Table 4 shows the effects of dietary supplementation of *Saccharomyces cerevisiae* fermentation product during late pregnancy and lactation on lactation performance of multiparous sows in a subtropical climate. Compared with L-THI group, sows from H-THI group had lesser individual piglet weight at weaning (*P* < 0.10), average daily gain of piglets (*P* < 0.05), and lactation average daily feed intake of sows (*P* < 0.05). Compared to sows fed the control diet, sows fed the SCFP diet had greater number of pigs weaned (*P* < 0.10), litter weight at weaning (*P* < 0.05), and average daily gain of piglets (*P* < 0.10). The number of piglets weaned was affected by THI × diet interaction (*P* < 0.05). The survival rate of piglets was not impacted by experimental treatments (*P* > 0.10).

**Table 4:** Effects of dietary supplementation of Saccharomyces cerevisiae fermentation product during late pregnancy and lactation on lactation performance of sows in a subtropical climate (Mean ± SE)^*^.

**Item**	**L-THI**		**H-THI**		***P*-value**		
	**Control diet**	**SCFP diet**	**Control diet**	**SCFP diet**	**THI**	**Diet**	**THI × Diet**
*N*	30	30	30	30			
No. of piglets per litter after cross-foster	10.4 ± 0.232	10.3 ± 0.211	9.6 ± 0.303	10.7 ± 0.244	0.436	0.126	0.263
Birth weight after cross-foster (kg)	15.6 ± 0.421	15.3 ± 0.451	14.4 ± 0.662	16.1 ± 0.571	0.742	0.183	0.072
Individual piglet weight after cross-foster (kg)	1.51 ± 0.032	1.48 ± 0.043	1.50 ± 0.042	1.50 ± 0.041	0.939	0.837	0.738
No. of pigs weaned/litter	9.14 ± 0.271^ab^	9.09 ± 0.262^ab^	8.77 ± 0.261^b^	9.86 ± 0.362^a^	0.484	0.073	0.049
Litter weight at weaning (kg)	45.6 ± 2.16	48.1 ± 1.54	42.4 ± 1.50	48.0 ± 2.14	0.382	0.032	0.405
Individual pigs weight at weaning (kg)	4.96 ± 0.153	5.32 ± 0.132	4.85 ± 0.121	4.91 ± 0.162	0.072	0.138	0.281
Piglet ADG (g/days)	165 ± 6	183 ± 5	159 ± 5	161 ± 7	0.029	0.098	0.191
Lactation ADFI (kg/days)	4.59 ± 0.074	4.66 ± 0.103	4.41 ± 0.091	4.37 ± 0.042	0.003	0.455	0.815
Survival rate of piglets (%)	88.0	87.9	88.4	92.0	0.611	0.984	NA

*ADG, average daily gain; ADFI, average daily feed intake; H-THI, high temperature-humidity index; L-THI, low temperature-humidity index, NA, not available*.

* *Results were expressed as mean and SE except for the survival rate of piglets as percentage. Values within a row with different superscripts differ significantly at P < 0.05*.

### Colostrum and Milk Composition

The effects of dietary supplementation of *Saccharomyces cerevisiae* fermentation product during late pregnancy and lactation on colostrum and milk composition of multiparous sows in a subtropical climate is summarized in Table 5. The compositions of colostrum and 21-days milk were not influenced by experimental treatments (*P* > 0.10). However, compared with L-THI group, sows from H-THI group had lesser protein percentage in 14-days milk (*P* = 0.107), while dietary SCFP supplementation or THI × diet did not affect the composition of 14-days milk (*P* > 0.10).

**Table 5:** Effects of dietary supplementation of *Saccharomyces cerevisiae* fermentation product during late pregnancy and lactation on colostrum and milk composition of sows in a subtropical climate (Mean ± SE).

**Item**	**L-THI**		**H-THI**		***P*-value**		
	**Control diet**	**SCFP diet**	**Control diet**	**SCFP diet**	**THI**	**Diet**	**THI × Diet**
*N*	6	6	6	6			
**Colostrum**							
Solids-not fat (%)	21.4 ± 2.61	21.2 ± 0.454	21.7 ± 0.921	20.0 ± 2.23	0.791	0.591	0.676
Protein (%)	8.15 ± 1.02	8.01 ± 0.193	8.20 ± 0.362	7.56 ± 0.881	0.780	0.574	0.780
Lactose (%)	11.6 ± 1.27	11.5 ± 0.232	11.7 ± 0.451	10.8 ± 1.15	0.780	0.604	0.663
Fat (%)	5.33 ± 0.723	5.62 ± 0.923	5.18 ± 1.59	6.29 ± 1.11	0.823	0.539	0.723
**14-days milk**							
Solids-not fat (%)	10.7 ± 0.224	10.4 ± 0.382	10.7 ± 0.411	10.5 ± 0.124	0.857	0.486	0.954
Protein (%)	3.94 ± 0.091	4.04 ± 0.164	3.75 ± 0.103	3.86 ± 0.044	0.107	0.340	0.971
Lactose (%)	5.98 ± 0.073	5.89 ± 0.234	5.74 ± 0.693	6.02 ± 0.133	0.722	0.580	0.248
Fat (%)	7.00 ± 0.614	7.43 ± 0.883	6.17 ± 0.691	6.07 ± 0.243	0.112	0.804	0.695
**21-days milk**							
Solids-not fat (%)	11.0 ± 0.422	11.3 ± 0.221	10.9 ± 0.293	11.1 ± 0.216	0.696	0.374	0.849
Protein (%)	4.04 ± 0.174	4.16 ± 0.083	4.03 ± 0.119	4.11 ± 0.084	0.788	0.350	0.896
Lactose (%)	6.07 ± 0.236	6.27 ± 0.118	6.05 ± 0.154	6.19 ± 0.121	0.773	0.356	0.844
Fat (%)	7.55 ± 0.811	6.92 ± 0.248	7.20 ± 0.464	7.16 ± 0.463	0.915	0.547	0.587

*H-THI, high temperature-humidity index; L-THI, low temperature-humidity index*.

### The Antioxidant Status in Serum of Sows

The effects of dietary supplementation of *Saccharomyces cerevisiae* fermentation product during late pregnancy and lactation on antioxidant status in serum of multiparous sows in a subtropical climate is displayed in Table 6. At 85-days post-breeding, i.e., the start of the feeding trial, antioxidant status including T-AOC, GSH-Px activity, SOD activity, GSH content, and MDA content was not different among experimental groups (*P* > 0.10). Compared with the L-THI group, sows from H-THI group had lesser T-AOC (*P* < 0.10) and SOD activity (*P* < 0.10) in serum at parturition, and lesser T-AOC (*P* < 0.10) and GSH-Px activity (*P* < 0.05) in serum at 14-days post-partum. However, compared with sows fed the control diet, sows fed SCFP diet had greater T-AOC in serum at parturition (*P* < 0.05). However, the GSH content (*P* < 0.05) and MDA content (*P* = 0.107) in serum of sows at parturition were affected by THI × diet interaction.

**Table 6:** Effects of dietary supplementation of *Saccharomyces cerevisiae* fermentation product during late pregnancy and lactation on antioxidant status in serum of sows in a subtropical climate (Mean ± SE).

**Item**	**L-THI**		**H-THI**		***P*-value**		
	**Control diet**	**SCFP diet**	**Control diet**	**SCFP diet**	**THI**	**Diet**	**THI × Diet**
*N*	6	6	6	6			
**85-days post-breeding**							
T-AOC (U/mL)	6.16 ± 1.97	7.30 ± 2.77	5.10 ± 1.17	7.23 ± 1.54	0.776	0.418	0.804
GSH-Px (U/mL)	1071 ± 32.7	1329 ± 169	1152 ± 64.0	1020 ± 127	0.322	0.578	0.100
SOD (U/mL)	98.6 ± 4.42	99.2 ± 5.76	88.0 ± 8.40	108 ± 7.05	0.866	0.146	0.171
GSH (mg/L)	2.07 ± 0.412	2.44 ± 0.891	2.07 ± 0.414	1.45 ± 0.429	0.406	0.823	0.406
MDA (nmol/mL)	2.17 ± 0.434	2.76 ± 0.699	1.99 ± 0.353	2.00 ± 0.274	0.334	0.542	0.542
**Parturition**							
T-AOC (U/mL)	2.71 ± 0.562	3.32 ± 0.301	1.83 ± 0.202	2.85 ± 0.344	0.091	0.043	0.586
GSH-Px (U/mL)	1455 ± 90.7	1480 ± 43.8	1432 ± 111	1346 ± 87.3	0.379	0.731	0.531
SOD (U/mL)	149 ± 3.58	145 ± 3.88	142 ± 4.47	132 ± 7.78	0.084	0.223	0.580
GSH (mg/L)	2.97 ± 0.571^a^	2.01 ± 0.363^ab^	1.89 ± 0.212^b^	2.98 ± 0.511^a^	0.904	0.904	0.033
MDA (nmol/mL)	1.49 ± 0.159	1.83 ± 0.141	1.71 ± 0.133	1.56 ± 0.142	0.861	0.583	0.107
**14-days post-partum**							
T-AOC (U/mL)	4.76 ± 0.619	4.71 ± 0.471	3.76 ± 0.314	4.17 ± 0.182	0.074	0.638	0.578
GSH-Px (U/mL)	1411 ± 73.9	1212 ± 96.2	1136 ± 56.0	1130 ± 61.5	0.028	0.182	0.207
SOD (U/mL)	116 ± 6.54	121 ± 6.71	111 ± 4.25	118 ± 5.08	0.531	0.277	0.929
GSH (mg/L)	4.38 ± 1.49	4.24 ± 1.27	5.77 ± 1.96	5.11 ± 1.28	0.468	0.802	0.867
MDA (nmol/mL)	1.43 ± 0.129	1.54 ± 0.110	1.55 ± 0.124	1.59 ± 0.081	0.423	0.455	0.717

*GSH, glutathione; GSH-Px, glutathione peroxidase; H-THI, high temperature-humidity index; L-THI, low temperature-humidity index; MDA, malondialdehyde; SOD, superoxide dismutase; T-AOC, total antioxidant capacity*.

### The Antioxidant Status of Colostrum and Milk

Table 7 gives the effects of dietary supplementation of *Saccharomyces cerevisiae* fermentation product during late pregnancy and lactation on antioxidant status in colostrum and milk of multiparous sows in a subtropical climate. Compared with the L-THI group, sows from the H-THI group had lesser SOD activity in colostrum (*P* < 0.05). Compared to sows fed the control diet, sows fed the SCFP diet had lesser MDA content in colostrum (*P* < 0.10) and 21-days milk (*P* < 0.05). The antioxidant status in colostrum, 14-days and 21-days milk were not impacted by THI × diet interaction (*P* > 0.10).

**Table 7:** Effects of dietary supplementation of *Saccharomyces cerevisiae* fermentation product during late pregnancy and lactation on antioxidant status in colostrum and milk of sows in a subtropical climate (Mean ± SE).

**Item**	**L-THI**		**H-THI**		***P*-value**		
	**Control diet**	**SCFP diet**	**Control diet**	**SCFP diet**	**THI**	**Diet**	**THI × Diet**
*N*	6	6	6	6			
**Colostrum**							
T-AOC (U/mL)	10.7 ± 2.67	11.3 ± 0.954	10.9 ± 1.47	12.3 ± 2.05	0.741	0.611	0.839
GSH-Px (U/mL)	84.3 ± 6.55	112 ± 21.8	84.6 ± 10.8	90.3 ± 10.4	0.443	0.238	0.430
SOD (U/mL)	167 ± 5.97	165 ± 1.66	154 ± 7.28	153 ± 2.42	0.025	0.796	0.910
GSH (mg/L)	5.41 ± 1.03	8.49 ± 1.09	6.74 ± 1.99	6.04 ± 0.69	0.671	0.379	0.165
MDA (nmol/mL)	2.07 ± 0.291	1.20 ± 0.214	1.80 ± 0.663	1.20 ± 0.312	0.719	0.068	0.715
**14-days milk**							
T-AOC (U/mL)	9.66 ± 0.383	12.8 ± 4.71	10.4 ± 1.22	18.8 ± 5.73	0.393	0.145	0.497
GSH-Px (U/mL)	76.6 ± 26.0	108 ± 14.3	66.5 ± 21.0	95.4 ± 27.6	0.619	0.202	0.950
SOD (U/mL)	146 ± 2.85	151 ± 4.15	154 ± 5.35	147 ± 3.37	0.665	0.893	0.161
GSH (mg/L)	65.3 ± 3.59	65.0 ± 15.1	58.1 ± 1.49	57.2 ± 27.8	0.642	0.972	0.985
MDA (nmol/mL)	5.24 ± 0.381	4.43 ± 0.393	5.13 ± 0.282	5.07 ± 0.346	0.471	0.299	0.303
**21-days milk**							
T-AOC (U/mL)	8.50 ± 3.66	11.3 ± 3.60	9.63 ± 1.46	7.89 ± 1.32	0.683	0.850	0.419
GSH-Px (U/mL)	99.8 ± 18.0	89.0 ± 4.47	97.1 ± 10.1	97.1 ± 13.3	0.832	0.671	0.671
SOD (U/mL)	139 ± 8.02	149 ± 9.68	146 ± 7.02	152 ± 5.98	0.562	0.312	0.789
GSH (mg/L)	88.5 ± 23.9	64.8 ± 8.88	72.1 ± 15.3	73.3 ± 7.48	0.797	0.473	0.427
MDA (nmol/mL)	3.48 ± 0.211	2.28 ± 0.283	3.06 ± 0.372	2.28 ± 0.421	0.524	0.008	0.524

*GSH, glutathione; GSH-Px, glutathione peroxidase; H-THI, high temperature-humidity index; L-THI, low temperature-humidity index; MDA, malondialdehyde; SOD, superoxide dismutase; T-AOC, total antioxidant capacity*.

## Discussion

### Reproductive Performance

The primary objective of this study was to investigate the effects of dietary supplementation of *Saccharomyces cerevisiae* fermentation product (SCFP) during late pregnancy and lactation on sow productivity, colostrum and milk composition, and antioxidant status of sows in a subtropical climate. In the present study, compared with sows from L-THI, sows from H-THI had lesser individual piglet birth weight (*P* < 0.10). It's reported that dietary supplementation of non-nutritive feed additives improved productive and physiological parameters of livestock (28, 29). However, dietary supplementation of SCFP did not affect reproductive performance of sows. Many researchers have demonstrated that yeast culture supplementation has no effect on reproductive performance, and their results are consistent (12, 22, 23, 30–32). Therefore, high THI impaired reproductive performance of sows, while dietary supplementation of SCFP did not affect reproductive performance.

### Lactation Performance

In the present study, compared with L-THI group, sows from H-THI group had lower individual piglet weight at weaning (*P* < 0.10), average daily gain of piglets (*P* < 0.05), and lactation average daily feed intake of sows (*P* < 0.05), which indicates that H-THI impaired lactation performance of sows in a subtropical climate. However, SCFP supplementation improved lactation performance of sows, indicated by the increased number of piglets at weaning (*P* < 0.10), litter weight at weaning (*P* < 0.05), and average daily gain of piglets (*P* < 0.10). It appears that SCFP supplementation relieved some of the adverse effects of high THI on lactation performance of sows. Kim et al. reported feeding SCFP (12 and 15 g/days during gestation and lactation, respectively) to sows during middle and late pregnancy and lactation increased litter weight gain by 6.9% (*P* < 0.01) (23). Kim et al. conducted another study involving 491 mixed-parity sows to explore the effects of feeding SCFP to sows during middle, late gestation and lactation on sow productivity, and they reported that SCFP supplementation increased litter weight gain for the multiparous sow (22). Similarly, Shen et al. (12) reported the effects of supplementation of SCPF during whole gestation and lactation on sow and litter performance, and found that feeding 12 (gestation) and 15 g/days SCFP (lactation) to sows had no effect on reproductive performance of sows, but improved litter weight at weaning (*P* = 0.068) and litter weight gain (*P* = 0.084) (12), which is in agreement with our results. Considering the supplementation dosage of yeast culture, the present study was 3 kg/t with 2.5–3.5 kg/d (gestation) and 4.37–4.66 kg/d (lactation) feed intake. Thus, the calculated yeast culture intake was 7.5–10.5 kg/d (gestation) and 13.11–13.98 kg/d (lactation), which is similar to the dosage of other reports (12, 22, 23). Regarding experimental duration, our study was conducted during late gestation and lactation, the studies of Kim et al. (22, 23) were done during middle, late gestation and lactation, while the study of Shen et al. (12) was conducted throughout the pregnancy and lactation. Even with different experimental durations, results were consistent. Therefore, it is reasonable to conclude that high THI impaired lactation performance of sows, while dietary supplementation of SCFP improved lactation performance of sows.

### Colostrum and Milk Composition

The nutritional composition and production of colostrum and milk is one of the main factors affecting the growth and development of nursing piglets (12). Many factors are impacting the composition and yield of colostrum and milk of dairy animals, such as animal breed, health status, environmental conditions and feeding program (33). Heat stress adversely affects the health of animals, and further negatively affects the composition and yield of colostrum and milk (33). Our results demonstrated that sows from H-THI had decreased protein percentage in 14-days milk (*P* = 0.107), which is probably mainly due to the decreased lactation feed intake of sows (*P* < 0.05). In our research, lactation feed intake and colostrum and milk composition were not improved by dietary supplementation of SCFP (*P* > 0.10). In agreement with our results, Shen et al. (12) reported that feeding SCFP to sows during the whole gestation and lactation did not impact the composition of colostrum and milk (12). Jang et al. directly fed live yeast to sows during pregnancy and lactation, and reported that live yeast had no beneficial effects on milk composition including fat, lactose, protein, solid-not-fat, and total solid (34). It is not clear how yeast culture can improve lactation performance of sows when the composition of colostrum and milk is unaffected. The most likely explanation is that yeast culture supplementation did not affect colostrum and milk composition of sows, but improved litter weight means through an increase of milk production (12). Therefore, H-THI negatively affected milk composition probably due to decreased lactation feed intake, while dietary supplementation of SCFP had no beneficial impact on colostrum and milk composition.

### The Antioxidant Status in Serum of Sows

Sows during the perinatal period suffer from high oxidative stress status due to greater metabolic activity (9, 11). Heat stress has been reported to induce reactive oxygen species (ROS) production due to the similarities in responses observed following heat stress compared to that occurring following exposure to oxidative stress (35). It's reported that mannan oligosaccharides supplementation can improve productivity and health status of layer chickens (36) and rabbits (37). SCFP includes mannan oligosaccharides. In the present study, H-THI decreased T-AOC (*P* < 0.10) and SOD activity (*P* < 0.10) in the serum of sows at farrowing, and lower T-AOC (*P* < 0.10) and GSH-Px activity (*P* < 0.05) in the serum of sows at 14-days post-partum, demonstrating the effects of H-THI on the serum antioxidant status in sows. In agreement with our results, Zhao et al. reported that heat stress aggravated oxidative stress of sows (38). However, sows fed the SCFP diet had higher T-AOC in serum at parturition (*P* < 0.05) compared with sows fed the control diet. This indicates that SCFP supplementation successfully increased the antioxidant status of sows. In agreement with our results, it was reported that yeast polysaccharides possess antioxidant function in both *in vivo* and *in vitro* models (39, 40). It was also reported that feeding yeast products enhanced serum and intestinal antioxidant indexes of weaned piglets (41). Yao et al. extracted water-soluble components from yeast culture, and found that it could protect intestinal mucosal cells of grass carp (*Ctenopharyngodon idella*), *in vitro*, from MDA-induced damage through enhancing cellular antioxidant capacity (42). Therefore, H-THI negatively affected the antioxidant status of sows, while SCFP supplementation improved the antioxidant status of sows.

### The Antioxidant Status in Colostrum and Milk

Colostrum and milk are very important and primary nutrient sources for newborns, and provide antioxidant protection for newborns in early life. Heat stress negatively affects antioxidant status in colostrum and milk, which indicates that heat stress may impact nursing babies. In the present study, compared with the L-THI group, sows from the H-THI group had lower SOD activity in colostrum (*P* < 0.05), which indicates that H-THI decreased the antioxidant status of colostrum. Compared to sows fed the control diet, sows fed the SCFP diet had lesser MDA content in colostrum (*P* < 0.10) and 21-days milk (*P* < 0.05), which indicates that SCFP supple-mentation increased the antioxidant status of colostrum and milk. Abuelo et al. reported that the redox balance of the colostrum had a significant effect on both calf oxidative status and passive immune transfer (43). The improved antioxidant status of colostrum and milk would help new-born piglets to enhance their poorly developed antioxidant system (44) and relieve upcoming weaning stress involving oxidative stress (45). Therefore, H-THI negatively affected the antioxidant status of colostrum, while SCFP supplementation improved the antioxidant status of colostrum and milk.

## Conclusions

In conclusion, H-THI negatively affected the productivity, milk composition, antioxidant status, and lactation feed intake of sows. Dietary supplementation of SCFP partially alleviated the adverse effects of H-THI, improved lactation performance and antioxidant status of sows without influencing reproductive performance and colostrum and milk composition.

## Data Availability Statement

All datasets generated for this study are included in the article/supplementary material.

## Ethics Statement

All animal protocols used in this study were approved by the South China Agricultural University Institutional Animal Care and Use Committee (SCAU-AEC-2010-0416).

## Author Contributions

WG and SZ: conceptualization. FC: methodology and supervision. MT and YuZ: software. JC, HS, and YuZ: validation. HQ: formal analysis. YL: investigation. YiZ: resources. HS: data curation. JC: writing original draft preparation and writing—review and editing. MT: visualization. SZ: project administration. WG: funding acquisition.

### Conflict of Interest

The authors declare that the research was conducted in the absence of any commercial or financial relationships that could be construed as a potential conflict of interest.
